# Phenomenon of hypocortisolism in individuals with obesity

**DOI:** 10.1016/j.cpnec.2025.100316

**Published:** 2025-09-05

**Authors:** Benedict Herhaus, Liza Mekschrat, Katja Petrowski

**Affiliations:** Medical Psychology and Medical Sociology, University Medical Center of the Johannes Gutenberg University Mainz, Mainz, Germany

**Keywords:** Obesity, Hypothalamic-pituitary-adrenocortical (HPA) axis, Cortisol, Basal hypocortisolism, Trier social stress test (TSST)

## Abstract

**Objective:**

Obesity has been associated with abnormalities of the hypothalamic-pituitary-adrenocortical (HPA)-axis. The aim of this study was to investigate patterns of cortisol stress reactivity and basal resting levels in individuals with obesity and healthy weight controls.

**Methods:**

Forty-seven individuals with obesity (BMI: 33.60 ± 4.09 kg/m^2^) and 47 age- and gender-matched healthy weight controls (BMI: 22.58 ± 1.89 kg/m^2^) underwent the Trier Social Stress Test (TSST) and a resting condition. Based on the salivary cortisol measurement during the TSST, individuals with obesity and the healthy weight controls were categorized into high/low cortisol reactivity group.

**Results:**

Obese low cortisol reactors demonstrated significantly lower basal cortisol resting levels compared to obese high cortisol reactors and healthy weight high/low cortisol reactors (*F*_(1,90)_ = 7.780, *p* ≤ .001, *η2* = .21). In individuals with obesity, we found an overlap between the high cortisol reactivity group and the high basal cortisol level group in the resting condition as well as between the low cortisol reactivity group and the low basal cortisol level group during the resting condition (*x*^*2*^ = 7.671, *df* = 1, *p* ≤ .01, Cramér's V = .40). This overlap could not be observed in the healthy weight controls.

**Conclusions:**

In conclusion, patterns of cortisol stress reactivity and basal resting levels were observed in the individuals with obesity but not in the healthy weight controls. The present data suggest that obesity may lead to the phenomenon of basal hypocortisolism.

## Highlights

• Lower basal cortisol resting levels in obese low cortisol reactors.
• Overlap of cortisol reactivity and resting levels in obesity, but not in healthy weight.
• Obesity may lead to the phenomenon of hypocortisolism.

## 1 Introduction

The hypothalamic-pituitary-adrenocortical (HPA)-axis as one of the major physiological stress systems is essential for responding to different internal and external stimuli to mediate allostasis [1]. Cortisol, the major stress hormone of the HPA-axis, has many functions throughout the body such as stress response, immune interactions [2], and drive of insulin resistance [3]. Therefore, when there is a short-term or long-term dysregulation of the HPA axis, it affects different areas in the body. In his allostatic load model McEwen [1] describes that repeated or chronic stress leads to an overexposure of the stress systems, including the HPA-axis resulting in pathophysiological consequences. Abnormalities of the HPA-axis are observed in different disorders like depression [4], insomnia [5], and chronic fatigue syndrome [6]. It should be noted that the dysregulation of the HPA-axis includes an over- and underactivation of the HPA-axis. Cortisol basal resting levels and cortisol stress reactivity are two important markers in the development of alterations of the HPA-axis [7].

There is also evidence of obesity-associated abnormalities of the HPA axis. However, it is unclear which comes first: obesity or HPA-axis dysregulation [8]. A meta-analysis demonstrated cortisol hyper- and hyporeactivity as well as hyper- and hypocortisolim in individuals with obesity compared to healthy weight controls [8,9]. Interestingly, the most consisting finding is lower morning cortisol levels in class I obesity compared to healthy controls [[10], [11], [12]]. It must be highlighted that an abnormal cortisol basal level is also a marker of a dysregulated HPA-axis [7]. For example, enhanced HPA-axis negative feedback loops resulted in lower basal cortisol levels in individuals with post-traumatic stress disorder compared to healthy controls [13]. Concerning the stress-induced cortisol response, the most consisting finding is higher cortisol response to acute stress in extreme obesity state [14] and obese women with central fat [15].

In individuals with obesity, alterations in the reductase activity of the transformation-enzyme 11β-HSD in the liver and in adipose tissue [16] increases expression of 11β-HSD1 [17,18], which could lead to a dysregulated cortisol activity through a disturbed feedback loop. A further possible reason for a change in the HPA-axis in obesity is the allostatic load model by McEwen [1]. His three types of allostatic load a) lack of adaptation, b) prolonged response and c) inadequate response can induce abnormalities of the cortisol metabolism with hyper- and hyporeactivity as well as hyper- and hypocortisolim. Individuals with obesity showed higher perceived chronic stress compared to individuals with healthy weight [19] and chronic stress is one of the major factors for change in the HPA-axis in obesity [20]. Hellhammer and Wade [21] postulated that in individuals with high chronic stress the development of a hyporeactive HPA-axis is the result of a preceding prolonged hyperactivated HPA-axis. Among highly stressed women with healthy weight there was an association between a higher BMI and lower cortisol basal levels [22]. Concerning the association of HPA-axis dysregulation and obesity, animal studies by Dallman et al. [[23], [24], [25]] showed that rats under high chronic stress chose overeating on palatable food as coping mechanism which over time led to weight gain and long-term blunted cortisol reactivity as well as low basal cortisol levels.

Cortisol measures of basal resting levels and stress reactivity are two important - to some extent independent - markers [7] in the development of alterations of the HPA-axis in obesity [8]. These forms of HPA-axis dysfunction might be explained by a different dysregulation of the glucocorticoid receptor (GR) and mineralcorticoid receptor (MR). Based on different neural correlates, the GR and MR have different roles in the HPA-axis regulation [26]. Basal activity of the HPA-axis is mainly regulated through MRs by proactive feedback while stress-induced activity is regulated through GRs by reactive feedback [26].

Based on our previous findings [27], which showed differences in cortisol activity − but not in stress reactivity − between individuals with obesity compared to healthy weight controls, the current study investigates patterns of cortisol stress reactivity and basal resting levels in individuals in a larger sample and adds an additional condition. Individuals with obesity and healthy weight controls underwent the standardized stress induction of the TSST and a resting condition. Based on evidence from a pharmacological challenge test showing lower baseline and stimulated total and salivary cortisol levels in individuals with obesity compared to healthy-weight controls [28], we hypothesized that the obese low cortisol reactivity group would show lower basal cortisol resting levels on the rest day compared to the obese high cortisol reactivity group and healthy weight high/low cortisol reactivity groups (hypothesis 1). A link of an abnormal functioning of the HPA-axis under basal resting and stress condition was observed in mental disorders like posttraumatic stress disorders and depression [13,29,30]. Therefore, we hypothesized an overlap of the high cortisol reactivity group during stress induction on the stress day and the high basal cortisol resting level group on the rest day and vice versa in the individuals with obesity but not in the healthy weight controls (hypothesis 2).

## 2 Material and methods

### 2.1 Study participants

For this study, forty-seven individuals with obesity ages 18–64 years (BMI: 33.60 ± 4.09 kg/m^2^) and forty-seven age- and gender-matched healthy weight controls (BMI: 22.58 ± 1.89 kg/m^2^) were recruited through advertisements in newspapers and online as well as flyers distributed in support groups. Volunteers were screened for inclusion criteria and medical history in advance, applying the Structured Clinical Interview (SCID: [31]) for the Diagnostic and Statistical Manual of Mental Disorders (DSM-IV: [32]) by telephone. Volunteers that reported an acute illness, chronic or mental disorders (including trauma-related disorders) in the present and past, medications that might influence the cortisol metabolism or substance intake, allergies or stressful life events as having occurred within the previous six months were excluded from participation. Participants were considered obese or of healthy weight as defined by the ICD-10 (obese: BMI >30 kg/m^2^; healthy weight: 18.5 kg/m^2^ < BMI >25 kg/m^2^). The study protocol was approved by the local Ethics Committee of the Landesärztekammer Rheinland-Pfalz, Germany (No#2018–13990). All participants gave written informed consent prior to their participation and were paid EUR 50.00 each in compensation for successful participation afterwards.

### 2.2 Procedures

All participants completed a resting condition as well as a stress condition in a laboratory of the institute on separate days over a time frame of one week. The time-schedule of both conditions was similar, but the testing sequence of the resting condition and the stress condition was randomized. Cortisol measurements were standardized with regard to their circadian rhythm by conducting all participant testing between 2:00 and 4:30 p.m. on weekdays. Female participants completed the stress test during their luteal phase to control for the influence of the menstrual cycle.

**Fig. 1:**
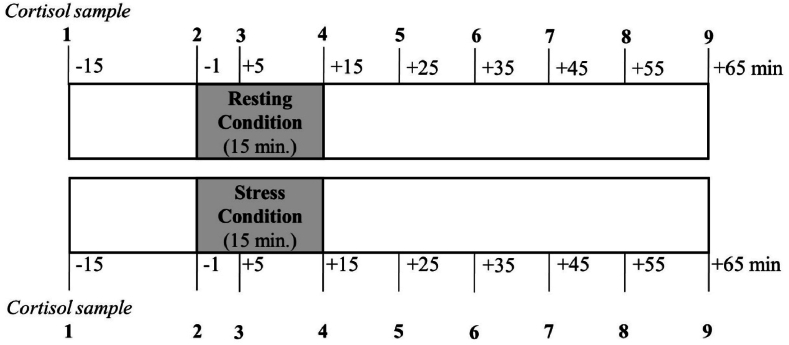
Overview of the cortisol measurement during resting/stress condition.

Both times, the study protocol was divided into a pre-stress, stress, and post-stress phase and took approximately 90 min to complete. Nine saliva samples were collected from each participant over the entire duration. After a stationary period of 30 min during which participants filled out questionnaires, the 15-min-pre-stress/resting phase began. During this preparatory phase, saliva samples were collected 15 min and 1 min before the start of each condition. As part of the stress condition, the participants underwent the TSST [33], separated into a preparation period, a mock-up job interview, and an arithmetic task, each lasting 5 min, thus 15 min in total (detailed description see Petrowski et al. [34]). During pre-stress/resting phase and during the resting condition, the participants were recommended to do some light reading (magazines made available or books brought by participants themselves, which are easy to read, do not produce deep thoughts or require concentration) to counteract any excessive arousal. During the stress condition and the resting condition, saliva samples were collected at five and 15 min after the start of each condition. Furthermore, the participants filled out the Primary Appraisal Secondary Appraisal (PASA) at the beginning of each condition and the visual analogue scale (VAS) immediately after the stress/resting condition to assess subjective stress perception. During the post-stress phase, five additional saliva samples were collected in time intervals of 10 min, while the participants had the opportunity to read magazines. A detailed overview of the cortisol measuring points can be found in Fig. 1.

### 2.3 Cortisol samples and analysis

The saliva samples were utilized to analyze cortisol concentration. For this purpose, the participants chewed on a cotton roll for 1 min, which was then placed in a salivette® (Sarstedt, Nümbrecht, Germany). After testing, the salivettes were refrigerated at 2–8 °C to protect them from exposure to heat and direct sunlight. For the laboratory analysis, the saliva was separated from the cotton roll via centrifugation and the salivary cortisol concentrations subsequently measured using the luminescence immunoassay (LIA) test method. This method proved to be robust and valid with an intra- and inter-assay coefficient of variation below 9.0 % [35].

### 2.4 Psychological assessments

The Trier Inventory for Chronic Stress (TICS: [36]) utilized in this study aims to assess chronic psychosocial stress that may have been experienced by the participants within the previous three months. The short version of nine items (TICS-9: [37]) used in this study represented the main dimensions of the long version (work overload, social overload, pressure to perform, work discontent, excessive demands at work, lack of social recognition, social tensions, isolation and chronic worrying). For this sample, internal consistency suggested a high homogeneity with Cronbach's α = .81 for the short version (TICS-9). To measure cognitive appraisal processes, the Primary Appraisal Secondary Appraisal (PASA: [38]) was applied at the beginning of each condition, a questionnaire that evaluates four cognitive appraisal processes named “threat”, “challenge”, “self-concept of own abilities”, and “control expectancy” concerning an anticipated situation on a six-point rating scale. Based on these four primary scales, two secondary scales “primary appraisal” ((threat + challenge)/2) and “secondary appraisal” ((self-concept of own abilities + control expectancy)/2) and the tertiary scale “stress index” (primary appraisal – secondary appraisal) are calculated. The primary scales “threat” (Resting Condition: .54; Stress Condition: .74), “challenge” (Resting Condition: .45; Stress Condition: .67), “self-concept of own abilities” (Resting Condition: .50; Stress Condition: .83), and “control expectancy” (Resting Condition: .81; Stress Condition: .85) showed satisfactory internal consistency (Cronbach's Alpha-coefficient) in the current sample except for the scales “threat” and “self-concept of own abilities” in the resting condition. The internal consistency values in the current sample for the tertiary scales “stress index” (Resting Condition: .62; Stress Condition: .75) demonstrated reasonable homogeneity. Subjective stress perception was furthermore assessed with the Visual Analogue Scale (VAS) immediately after the stress and resting condition and consists of eight items (for example “I found the situation challenging”). The VAS is a 10 cm line with anchor statements on the left (“not at all”; 0 cm) and on the right (“very much”; 10 cm). The VAS is a scale that has proven to be a valid instrument [39]. In the current sample the Cronbach's Alpha-coefficient for the VAS was .60.

### 2.5 Statistical analysis

For the specification of the cortisol stress reactivity in the TSST and cortisol concentration during the resting condition of the participants, the area under the curve with respect to ground (AUC_G_) and increase (AUC_I_) was calculated with formulas by Pruessner et al. [40]. Subgroups of high and low cortisol reactivity (AUC_I_) as well as high and low basal cortisol resting level (AUC_G_) were formed separately for both groups (individuals with obesity/healthy weight controls) by a median split of the AUC_I_ of the stress condition and AUC_G_ of the resting condition. The 47 individuals with obesity were divided into a high (AUC_I_ > 3.47 nmol/l∗min, *n* = 24) and a low (AUC_I_ < 3.47 nmol/l∗min, *n* = 23) cortisol reactivity group as well as a high (AUC_G_ > 3.28 nmol/l∗min, *n* = 24) and a low (AUC_G_ < 3.28 nmol/l∗min, *n* = 23) basal cortisol resting level group. The 47 healthy weight controls were also categorized into a high (AUC_I_ > 3.07 nmol/l∗min, *n* = 24) and a low (AUC_I_ < 3.07 nmol/l∗min, *n* = 23) cortisol reactivity group as well as a high (AUC_G_ > 5.04 nmol/l∗min, *n* = 24) and a low (AUC_G_ < 5.04 nmol/l∗min, *n* = 23) basal cortisol resting level group. The data of the cortisol concentration and derived cortisol parameters were analyzed according to the normality of distributions and were, in case of not normally distributed data, subjected to logarithm naturalis +1 transformations. All statistical analyses were conducted using SPSS Statistics version 27 (IBM, Chicago, IL, USA).

Firstly (1), the success of the stress induction was checked by cortisol response and subjective stress appraisal (questionnaire PASA & VAS). For the cortisol response, the area under the curve with respect to increase (AUC_I_) and the delta between peak and baseline (Δ Peak-Base) were calculated. All parameters (PASA, VAS, Cortisol-AUC_I_, & Cortisol Δ Peak-Base) were analyzed by two-factorial MIXED ANOVA for repeated measurements with the within-factor condition (stress vs. resting) and between-factor group (obese vs. healthy weight).

Secondly (2), the cortisol AUC_G_ (resting condition) was analyzed by two-way ANOVA with the between-factor BMI-class (obese vs. healthy weight) and between-factor cortisol stress reactivity group (high vs. low cortisol reactivity group). Additionally, differences between the four groups of obese high cortisol reactivity group, obese low cortisol reactivity group, healthy weight high cortisol reactivity group, and healthy weight low cortisol reactivity group in the cortisol AUC_G_ (resting condition) were tested by independent Student t-tests with Bonferroni-Holm correction.

Thirdly (3), the Chi-square test was performed to test the overlap between the subgroups of low/high cortisol reactivity and low/high basal cortisol resting level in the individuals with obesity and the healthy weight controls.

## 3 Results

### 3.1 Sample characteristics

In Table 1, the characteristics of the two groups (*N* = 47 each) are depicted. There were no significant differences between the two groups for age, gender, number of smokers, and use of oral contraceptives (*p's* > .05). However, it must be considered that the individuals with obesity demonstrated higher scores in the perception of chronic stress (TICS-9) compared to the healthy weight controls (*t* (92) = 2.036, *p* = .045, *d* = .42). The four subgroups, categorized by BMI-class and cortisol reactivity, did not differ in age, gender, number of smokers, and use of oral contraceptives (*p's* > .05).

**Table 1:** Characteristics of the participants regarding matching criteria.

	Individuals with obesity				
	Total Sample	Individuals with obesity	Individuals with healthy weight	*t/x*^2^	*p*
Females, *n (%)*	48 (51.1)	24 (51.1)	24 (51.1)	0	1.00^a)^
Age, *M (SD)*	37.07 (13.07)	37.91 (13.10)	36.23 (13.13)	.621	.54^a)^
BMI, *M (SD)*	28.09 (6.38)	33.60 (4.09)	22.58 (1.89)	16.779	≤.001^a)^ (*d* = 3.47)
Smokers, *n (%)*	10 (10.2)	3 (6.4)	7 (14.9)	1.790	.18^b)^
Contraceptives *n* (*%* of females)	10 (21.3)	4 (16.7)	6 (25.0)	.505	.48^b)^
TICS-9, *M (SD)*	10.81 (5.36)	11.92 (5.50)	9.70 (5.03)	2.036	.045^a)^ (*d* = .42)

Note. BMI - body mass index; d - Cohen; M − Mean; SD - Standard Deviation; TICS - Trier Inventory of Chronic Stress; a) Independent Student t-test b) Chi-square test.

### 3.2 Stress induction

In the individuals with obesity and the healthy weight controls, there was a significant effect of time with higher values in the stress condition in cortisol AUC_I_ (*F*_(1,92)_ = 80.593, *p* ≤ .001, *η2* = .47) and cortisol Δ Peak-Base (*F*_(1,92)_ = 76.131 *p* ≤ .001, *η2* = .45). ANOVA results indicated no significant time × group interaction effect in AUC_I_ and Δ Peak-Base (see Table 2). There was no difference in the stress-induced cortisol response between individuals with obesity and the healthy weight controls (AUC_I_: t (92) = −.158, *p* = .88; Δ Peak-Base: t (92) = .787, *p* = .97). There was also no time x group x gender effect in AUC_I_ (*p* = .42) and Δ Peak-Base (*p* = .25). For all participants, a significant difference between the resting condition and the psychosocial stress condition could be observed in the PASA primary scales threat (*F*_(1,92)_ = 261,240, *p* ≤ .001, *η2* = .74), challenge (*F*_(1,92)_ = 140,506, *p* ≤ .001, *η2* = .60), self-concept (*F*_(1,92)_ = 34,676, *p* ≤ .001, *η2* = .27), on PASA tertiary scale stress-index (*F*_(1,92)_ = 139,000, *p* ≤ .001; *η2* = .60), and on the VAS (*F*_(1,92)_ = 90,535, *p* ≤ .001; *η2* = .50), indicating that the participants perceived greater anticipatory and acute stress during the psychosocial stress condition compared to the resting condition. There was no time × group interaction effect in all PASA scales and in the VAS (see Table 2).

**Table 2:** Subjective appraisal and hormonal response with respect to conditions in individuals with obesity and healthy weight controls.

	Individuals with obesity*(n=47)*		Individuals with healthy weight*(n=47)*		MIXED ANOVA		MIXED ANOVA	
					Condition Effect		Condition∗Group Effect	
Subjective Appraisal	Resting Condition	Stress Condition	Resting Condition	Stress Condition	*F*	*p*	*F*	*p*
PASA – Threat, M (SD)	1.64 (.71)	3.30 (.95)	1.26 (.44)	3.09 (.95)	261.240	≤.001∗∗	.663	.42
PASA – Challenge, M (SD)	2.75 (.81)	4.10 (.91)	2.68 (.94)	4.27 (.80)	140,506	≤.001∗∗	.930	.34
PASA – Self-concept, M (SD)	4.40 (.90)	3.91 (.90)	4.64 (.80)	3.93 (1.10)	34.676	≤.001∗∗	1.086	.30
PASA – Control expectancy,	3.90 (1.25)	4.63 (.79)	3.90 (1.26)	4.36 (1.18)	16.919	.16	.915	.34
M (SD)								
PASA - Stress index, M (SD)	−1.96 (1.19)	−.57 (1.16)	−2.30 (.88)	−.47 (1.16)	139.000	≤.001∗∗	2.716	.10
VAS, M (SD)	41.16 (12.11)	55.68 (15.80)	40.69 (10.84)	55.48 (11.34)	90.535	≤.001∗∗	.007	.93
**Hormonal Response**								
AUC_I_, M (SD)	.41 (83.85)	350.23 (368.19)	−6.58 (99.6)	337.95 (383.74)	80.593	≤.001∗∗∗	.005	.95
Δ Peak-Base, M (SD)	1.76 (2.23)	11.09 (10.21)	1.93 (2.18)	11.01 (11.02)	76.131	≤.001∗∗∗	.014	.91

### 3.3 Association between cortisol stress reactivity and cortisol resting levels

**Fig. 2:**
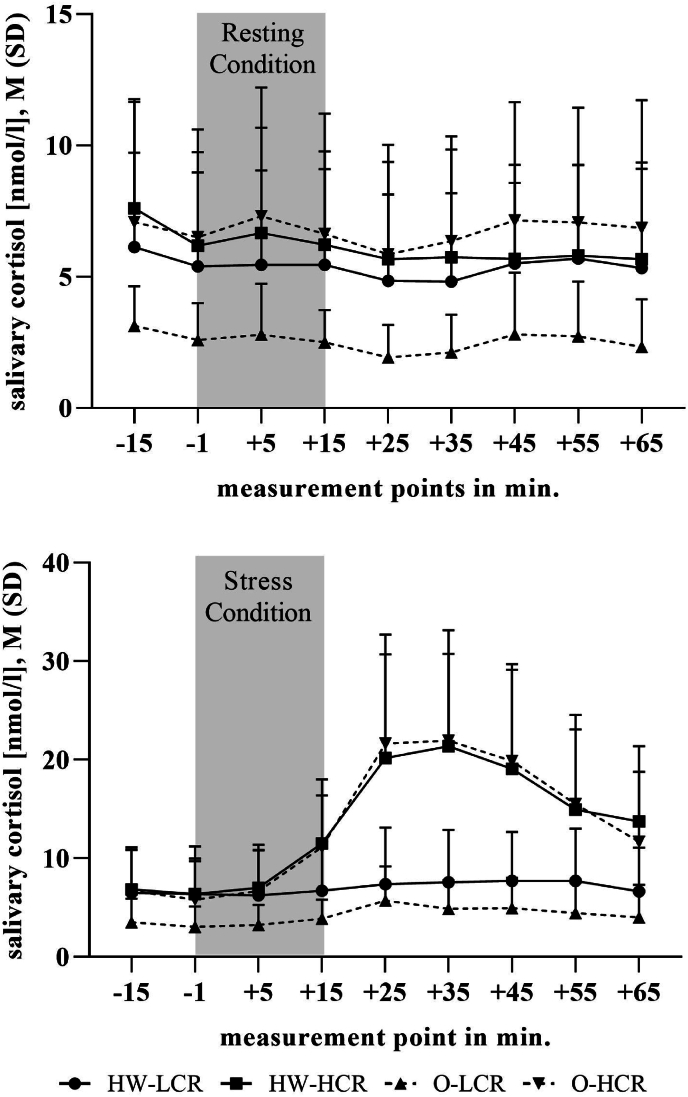
Salivary concentrations (M + SD) during resting and stress condtion in obese and healthy weight low/high cortisol reactores.

**Fig. 3:**
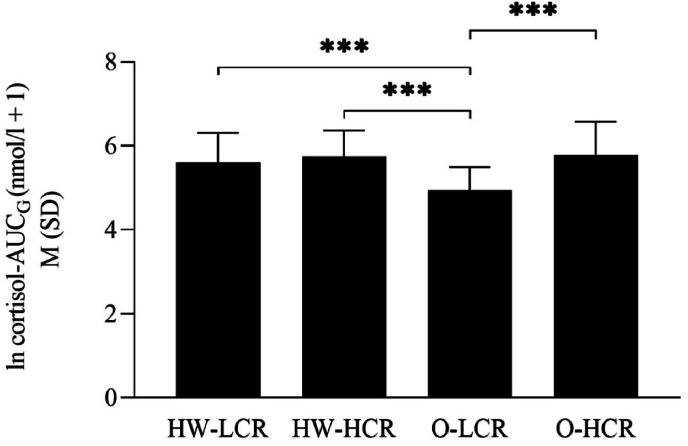
Cortisol -AUC_G_ of the resting condition in obese and healthy weight low/high cortisol reactivity groups.

Concerning the cortisol-AUC_G_ of the resting condition, ANOVA demonstrated significant differences between the factor BMI-class (obese vs. healthy weight) (*F*_(1,89)_ = 5.528, *p* = .021, *η2* = .06) and factor cortisol stress reactivity group (high vs. low cortisol reactivity group) (*F*_(1,89)_ = 12.040, *p* ≤ .001, *η2* = .12). The interaction effect BMI-class x cortisol stress reactivity group revealed a significant result (*F*_(1,89)_ = 6.319, *p* = .014, *η2* = .07). As shown in Fig. 2, Fig. 3, and Supplemental Fig. 1, obese low cortisol reactors showed significantly lower cortisol-AUC_G_ of the resting condition to obese high cortisol reactors (*t* (29.29) = −4.248, *p* ≤ .001, *d* = −1.22), healthy weight high cortisol reactors (*t* (32.84) = −4.264, *p* ≤ .001, *d* = −1.23), and healthy weight low cortisol reactors (*t* (32.24) = −3.664, *p* ≤ .001, *d* = −1.08). To further examine the association between basal cortisol levels and cortisol reactivity beyond the median-split subgrouping, we conducted separate regression analyses for individuals with obesity and healthy-weight controls. In individuals with obesity, cortisol AUC_G_ from the resting condition significantly predicted cortisol AUC_I_ from the stress condition, (*R*^*2*^ = .35, *F*_(1,45)_ = 24.06, *p* ≤ .001). In contrast, this association was not significant in healthy-weight controls, (*R*^*2*^ = .03, *F*_(1,45)_ = 1.53, *p* = .22). In addition, in the individuals with obesity there was a significant overlap of the subgroup high cortisol reactivity group in the stress induction and high basal cortisol level group during resting condition as well as between the low cortisol reactivity group in the stress induction and the low basal cortisol group during the resting condition (*x*^*2*^ = 7.671, *df* = 1, *p* ≤ .01, Cramér's V = .40). No significant overlap could be overserved in the subgroups of the healthy weight controls.

## 4 Discussion

This study investigated patterns of cortisol stress reactivity and basal resting levels in individuals with obesity and healthy weight controls. As expected, the obese low cortisol reactivity group showed significantly lower basal cortisol resting level in contrast to the obese high cortisol reactivity group and the healthy weight high/low cortisol reactivity group. Furthermore, our findings showed in individuals with obesity an overlap between the high cortisol reactivity group in the stress induction and the high basal cortisol level group during the resting condition as well as between the low cortisol reactivity group in the stress induction and the low basal cortisol level group during the resting condition. In healthy weight individuals, there was no overlap between the high cortisol reactivity group in the stress induction and the high basal cortisol level group during the resting condition or between the low cortisol reactivity group in the stress induction and the low basal cortisol level group during the resting condition.

The present finding of no association between patterns of cortisol stress reactivity and basal resting levels in healthy weight individuals is in line with a previous study of healthy male participants [7]. By contrast, similar studies have demonstrated a link of an abnormal functioning of the HPA-axis under basal resting and stress condition in mental disorders like posttraumatic stress disorders and depression [13,29,30]. The present study is one of the first demonstration that in individuals with obesity high cortisol stress reactivity was linked to high cortisol basal resting levels and vice-versa. In line with this, laboratory study by Tomiyama et al. [22] showed an association between a higher BMI, lower cortisol stress reactivity and lower cortisol basal levels in women with healthy weight. Moreover, previous research indicates that patterns of basal HPA-axis activity are associated with health-relevant outcomes: healthier profiles have been linked to stronger initial cortisol and IL-6 reactivity and greater habituation across repeated stress exposures [41], and evidence that a stronger preceding cortisol release can attenuate subsequent physiological and perceived stress responses [42]. Consequently, the basal state of the stress system at the time of the stressor plays a critical role in modulating endocrine and emotional stress responses, as demonstrated by findings that pharmacologically increasing cortisol before the TSST reduces subjective stress [43,44].

This association of patterns of cortisol stress reactivity and basal resting activity in obesity might be explained by the altered obesity-associated metabolism and his effect on receptor function. There is evidence of an abnormal functioning of the HPA-axis with disturbed feedback loop through an increased adipose tissue with increased expression of the transformation-enzyme 11β-HSD1 [17,18]. We speculate that obesity may lead to stronger feedback loop on the HPA-axis resulting in lower basal cortisol resting levels. This is supported by the lower basal resting levels in obese low cortisol reactors compared to healthy weight high cortisol reactors and healthy weight low cortisol reactors. With regard to the different roles of GR and MR in the HPA-axis regulation, one possible mechanism might be diminished sensitivity of the MR. The MR mainly regulated the basal activity of the HPA-axis but also involved in the stress response with the GR [45]. There is evidence from animal and cell culture studies, that reduced MR plays a pivot role in the development of an abnormal functioning of the HPA-axis in obesity [46,47].

A further possible explanation for the hypocortisolism in obesity might be the effect of high chronic stress on the HPA-axis. Individuals with obesity reported significantly higher levels of perceived chronic stress on the TICS-9 than healthy-weight controls, consistent with previous findings [19,20]. According to the allostatic load model proposed by McEwen [1], repeated or prolonged activation of the HPA-axis due to chronic stress can lead to an eventual downregulation of the system, resulting in blunted cortisol reactivity and lower basal cortisol levels—a phenomenon often referred to as hypocortisolism. This process is thought to represent a physiological adaptation to protect tissues from the harmful effects of prolonged glucocorticoid exposure [48,49]. In addition, chronic stress has been linked to changes in peripheral glucocorticoid sensitivity, with direct effects on central nervous system and HPA-axis circuits, including the amygdala, hippocampus, and peripheral tissue glucocorticoid receptors [50], which may further contribute to altered basal HPA-axis activity in obesity.

Our data provide further support for a possible phenomenon of basal hypocortisolism in individuals with obesity [9]. The basal hypocortisolism in obesity affects different functions throughout the body. Cortisol release is an important regulator of the sympathetic nervous systems through inhibitory effect on the catecholamine synthesis, especially during stress situations. In case of basal hypocortisolism, down-regulation of the inhibitory feedback activities of cortisol results in an over-secretion of catecholamines with an overactivity of the sympathetic nervous system [48]. An overactivity of the SNS could be observed in individuals with obesity [51] which is also a risk factor of cardiovascular diseases in obesity [52]. Furthermore, there is evidence of an overactivity of the immune system in stress-related disorders with hypocortisolism [53]. Glucocorticoids are described as one important determinant in the regulation of the immune system with anti-inflammatory effects [54]. Therefore, low cortisol levels may result in an increase in inflammatory such as IL-6. For example, Rexrode et al. [55] found that obesity is strongly associated with increased pro-inflammatory cytokine concentrations of IL-6 and CRP.

The strengths of this study are: the age- and gender-matched groups of the individuals with obesity and the healthy weight controls, the control of confounding factors within the cortisol measurement (e.g., menstrual cycle and circadian rhythm), and the long measuring period (90 min) with frequent measurements points during the two conditions: stress and resting. The main limitation of this study is the missing longitudinal design which is necessary to detect possible changes in the HPA-axis in individuals with obesity over a period of time. With regard to higher cortisol response to acute stress in extreme obesity state [14] and obese women with central fat [15], another limitation is the non-assessment of further information about the obesity profile (e.g., determination of abdominal/visceral obesity, duration of obesity exposure). Despite the use of a median split which, according to Maxwell and Delaney [56], has some limitations and reduces power to find an effect, significant effects were observed in our study. In addition, previous neuroimaging evidence suggests that BMI is associated with increased brain responses and greater negative affect after stress, with individual response profiles being significantly related to BMI in females but not in males [57]. Future studies should therefore examine whether the associations between basal cortisol, stress reactivity, and obesity differ by sex, ideally using sufficiently powered samples to allow sex-stratified analyses. An additional limitation is the small cell sizes in the distribution analysis. With regard to the effect of basal hypocortisolism on the immune system and the SNS, the non-assessment of biomarkers like cytokines or catecholamines is a limitation.

## 5 Conclusion

In conclusion, patterns of cortisol stress reactivity and basal resting levels were observed in the individuals with obesity but not in the healthy weight controls. The present cross-sectional findings suggest that obesity may lead to the phenomenon of basal hypocortisolism.

## CRediT authorship contribution statement

**Benedict Herhaus:** Writing – original draft, Visualization, Validation, Software, Resources, Project administration, Methodology, Investigation, Funding acquisition, Formal analysis, Data curation, Conceptualization. **Liza Mekschrat:** Writing – review & editing, Writing – original draft, Supervision. **Katja Petrowski:** Writing – review & editing, Writing – original draft, Supervision.

## Funding and support

This study was funded by the DFG-project 'Comparison of the chewing behavior of patients with obesity and healthy weight controls under resting and stress conditions' (Project-number: 276734837).

## Declaration of competing interest

The authors declare that they have no known competing financial interests or personal relationships that could have appeared to influence the work reported in this paper.
