# Supplementary figures and images for: Phenomenon of hypocortisolism in individuals with obesity
Source: Compr Psychoneuroendocrinol. 2025 Sep 5;24:100316. doi: 10.1016/j.cpnec.2025.100316 (PMC12452850; doi:10.1016/j.cpnec.2025.100316)

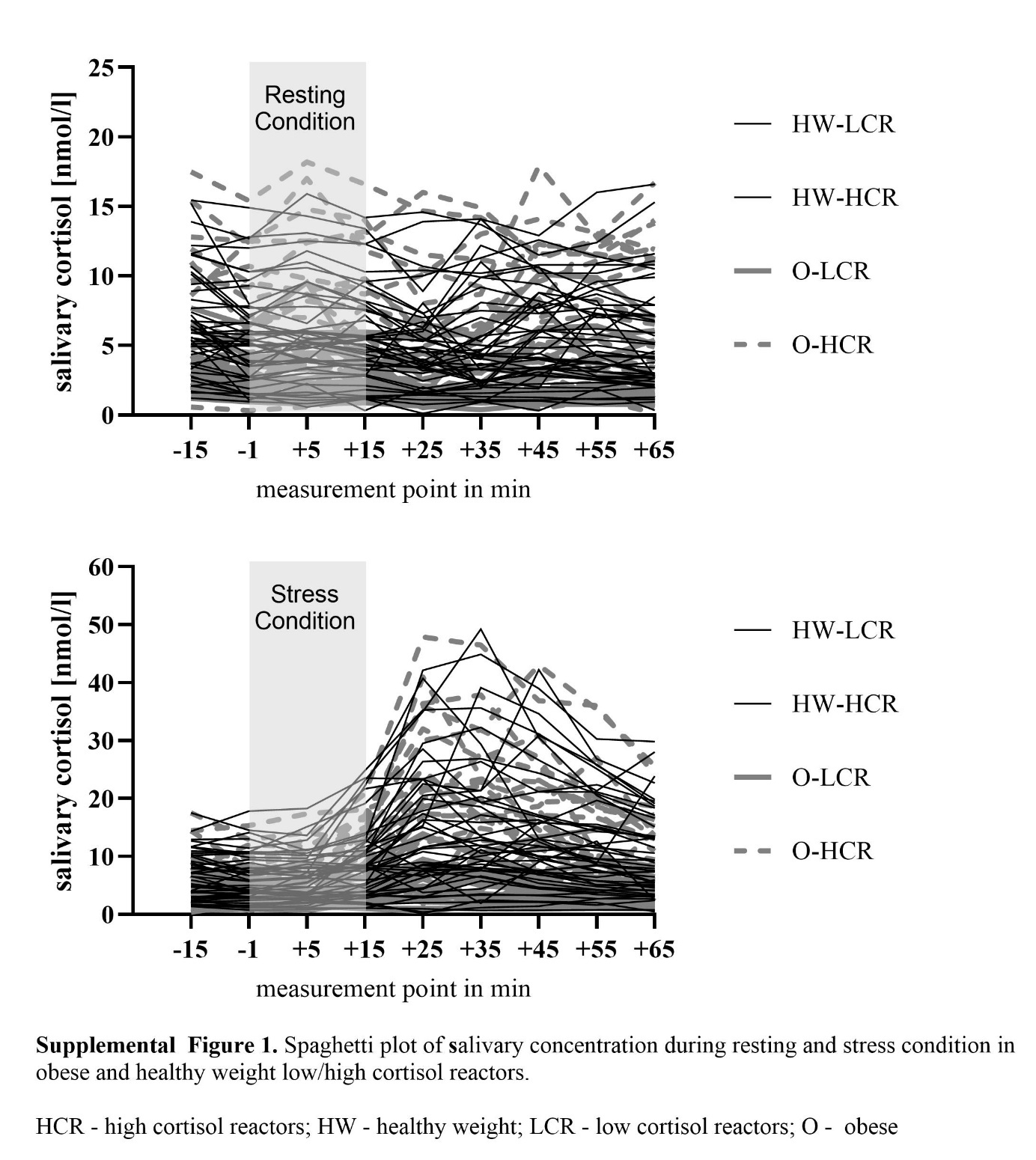

Supplement: Multimedia component 1 [file mmc1.docx]
